# Left Ventricular Diastolic Function in Nigerian Patients with Essential Hypertension: A Retrospective Study to Compare Angiotensin Converting Enzyme Inhibitors, Calcium Channel Blockade or Their Combination

**DOI:** 10.1111/j.1753-5174.2008.00005.x

**Published:** 2008-07

**Authors:** Olufemi E Ajayi, Anthony O Akintomide, Adegboyega Q Adigun, Adesuyi A L Ajayi

**Affiliations:** * Department of Medicine, Division of Cardiology, College of Health Sciences, Obafemi Awolowo UniversityIle-Ife, Nigeria; † Department of Medicine, Reading HospitalReading, Philadelphia, PA, USA; ‡ Division of Hypertension and Clinical Pharmacology, Department of Medicine, Baylor College of MedicineHouston, TX, USA

**Keywords:** Essential Hypertension, Diastolic Function, Left Ventricular Hypertrophy, Blacks, ACE Inhibitors, Calcium Antagonists, Doppler Echocardiography

## Abstract

**Background:**

Hypertension in blacks imposes a greater left ventricular hypertrophy, and accelerated heart failure onset. We evaluated and compared the echocardiographically determined systolic and left ventricular diastolic functional indices in Nigerian hypertensive patients, associated with the chronic use of ACE inhibitors, Calcium channel blockers (CCB) or their combinations.

**Methods:**

Ejection fraction -EF, intraventricular relaxation time (IVRT), E/A peak velocity ratio, E wave deceleration time] as well as the left ventricular mass index (LVMI) was undertaken among 41 Nigerian patients with essential hypertension only, on treatment for 4–6 months prior. The 41 patients (aged 59 ± 10 years, 40% females) were divided into three groups; those receiving (i) ACE inhibitors; or (ii) CCB or (iii) combination of ACEI and CCB. All the three groups had a background of diuretic treatment for optimal blood pressure control.

**Results:**

There were no statistically significant differences in the mean LVMI or sitting blood pressure between treatment groups. E/A ratio for ACEI treatment was 1.06 ± 0.44, CCB 0.74 ± 0.19, and for ACEI + CCB 0.87 ± 0.26 (*F* = 3.29, *P* = 0.048 anova). The 95% confidence interval for the E/A ratio on ACEI was 0.8 to 1.33. The A wave duration time integral (AVVTi) were all abnormally large, but showed a significant between treatment group difference (*P* = 0.037, anova). The values were 21.9 ± 4.7 for ACEI, 25.3 ± 6.3 for CCB, and least at 20.1 ± 3.6 cm for the ACE + CCB combination. Similarly, the IVRT was lowest and <100 ms with ACEI + CCB being 93 ± 18 ms, ACEI 115 ± 23 ms, and CCB being 117 ± 22 ms (*F* = 4.92, *P* = 0.01, anova). The 95% CI for IVRT on ACEI + CCB was 82 to 104 ms. There were no between treatment group differences in systolic contractility, (fractional shortening or EF).

**Conclusions:**

The results indicate that use of an antihypertensive drug regime inclusive of an ACE inhibitor (±CCB) may be associated with greater salutary effect on indices of diastolic function, (E/A > 1, lower AVVTi, IVRT < 100 ms) even in the presence of an equivalent effect on systolic function and blood pressure.

## Introduction

Essential hypertension remains a global disease, which has the highest prevalence in blacks in America [1] as well as in Sub-Saharan African nations [2]. The target organ complications of hypertension are also both more frequent and severe among blacks. Hypertension remains the leading cause of cardiovascular disease [3], congestive heart failure [4], cerebrovascular and end-stage renal disease [5,6] as well as sudden cardiac death and total mortality among black patients [7–9]. The dismal effects of hypertension on the heart of blacks in Africa and America has been previously described using echocardiography and pathologic samples [10–12].

It has been reported that at a given level of blood pressure control, blacks exhibit a more impaired function and geometric distortions than Caucasians [13]. Ethnic differences both in the progression of systolic and diastolic dysfunction, and mortality have been reported, with a worse outcome in blacks [14–17]. The mechanisms of these ethnicity related differences are not known with certainty, and socioeconomic factors may contribute. However, there is a strong suggestion of a genetic predisposition of blacks to accelerated and severe cardiac dysfunction, based on their increased prevalence of homozygotes for the polymorphisms of the adrenergic receptors Beta-1 and Alpha-2C [18]. The imperative for pharmacotherapeutic agents or regimens that control blood pressure and retard or prevent target organ damage of hypertension, especially in blacks is thus clearly obvious. While there are reports of such studies in Caucasians and other populations [19–21], the impact of antihypertensive treatment on echocardiographically determined cardiac systolic or diastolic function in Sub-Saharan blacks is sparse. Further, although we previously reported on a more rational treatment of hypertension in a Nigerian tertiary center [22], it is not clear if the profile of blood pressure control will translate to cardiac organ protection in this “at risk” population.

We therefore conducted an exploratory retrospective study to evaluate and compare the echocardiographically determined, cardiac systolic and diastolic functional indices, associated with different antihypertensive treatment regimens in Nigerian patients with essential hypertension who have received prolonged drug therapy.

## Materials and Methods

A total of 102 patients with essential hypertension with echocardiographic records were available for review. None of these patients had a history of concurrent diabetes mellitus, hypertensive heart failure (or of its history), renal disease, significant valvular heart disease, or angina pectoris which were exclusion criteria. Patients were also excluded if they had poor echocardiographic recordings, or if they were on a different set of antihypertensive drug regimen, other than ACEI, CCB, or their combination, with a diuretic background. Patients on thiazide diuretics alone or frusemide alone were also excluded. Of the 102 patients, 41 fulfilled the inclusion criteria of being on the same indicated antihypertensive pharmacotherapeutic regime for at least 3–4 months, before the echocardiographic study, and in sinus rhythm by EKG. Their blood pressures were also well controlled, and there were no between group statistical differences in the systolic or diastolic blood pressures (See Table 1). The local hospital ethics and research committee gave approval to conduct this retrospective review of the clinical and echocardiographic impact of the treatments, as previously reported by us [22].

**Table 1:** Demographic and echocardiographic dimensions, systolic and diastolic function in Nigerians with essential hypertension on long term drug therapy with ACE—inhibitors, calcium antagonists or their combination. Mean (SD)

	ACE-inhibitor regimen	Calcium antagonist regimen	ACE-inhibitor + Calcium antagonist
*n*	13	12	16
Age(years)	58 (12)	64 (9)	55 (11)
Sex (M/F)	6/7	7/5	11/5
Systolic BP (mmHg)	143 (11)	138 (9)	141 (12)
Diastolic BP (mmHg)	88 (6)	89 (10)	84 (7)
Heart rate (min^−1^)	81 (14)	82 (10)	83 (13)
LVMI (g/m^2^)	125 (35)	144 (37)	149 (41)
RWT	0.52 (0.18)	0.50 (0.11)	0.61 (0.13)
LVEF (%)	79 (11)	76 (10)	72 (10)
Fractional shortening (%)	38 (8)	39 (9)	36 (7)
IVRT (ms)	115 (23)	117 (22)	93 (18)*
E/A ratio	1.06 (0.44)	0.75 (0.19)	0.87 (0.26)*
AVVTi (cm)	21.9 (4.7)	25.3 (6.3)	20.1 (3.6)*

* *P* < 0.05 anova between groups. AVVTi = A wave velocity time integral.

BP = sitting blood pressure; RWT = relative wall thickness; LVMI = left ventricular mass index; LVEF = left ventricular ejection fraction; IVRT = Intraventricular relaxation time; E/A ratio = ratio of early to atrial peak transmitral wave velocities.

An M–mode and 2 dimensional echocardiographic study was undertaken using an a Siemens sonoline G60S ultrasound machine with a 2.5 Mhz, 3.5 Mhz, probes, 4.2 Mhz probe for Doppler studies. Cardiac dimensions and left ventricular mass and mass index were calculated using the Penn equation [23]. Systolic ejection fraction and fractional shortening were calculated using standard equations. Diastolic function (Early and Atrial peak velocities and their ratios E/A velocity ratio, the A wave velocity time integral AVVTi, and the intraventricular relaxation time IVRT, from the closure of the aortic valve to the opening of the mitral valve) was measured using pulse- wave Doppler in which the sample volume was placed at the tips of the mitral valve leaflets in the apical 4 chamber view [24]. The IVRT was measured as the time interval between the end of the LV outflow and the start of LV inflow, as indicated by simultaneous registration of inflow and outflow signals by the high frequency- pulsed–wave Doppler. These diastolic parameters were chosen because they have been shown to be abnormally prolonged or altered in essential hypertension and are correlated to the level of blood pressure [11,17,20].

The records of eligible patients (N = 41) were then sub-divided to three groups according to their therapeutic regime. Group A (N = 13) were patients treated with angiotensin converting enzyme inhibitors; enalapril 5–10 mg daily, or lisinopril 5–20 mg daily with concurrent thiazide diuretic treatment. Group C (N = 12) received calcium channel blockers; amlodipine 5–10 mg daily or rarely sustained release nifedipine 20 mg daily, with thiazide diuretic (12.5–50 mg) to achieve better blood pressure control. Group A + C (N = 16), received a combination of angiotensin converting enzyme inhibitors and calcium channel blockers with a background of thiazide diuretics (mostly hydrochlorothiazide 12.5–25 mg daily). Patients in the three groups had been on these antihypertensive medications for at least three months, prior to the echocardiographic record. The clinical, demographic and echocardiographic data in the three pharmacotherapeutic groups of treated hypertensives are summarized in Table 1.

Data is expressed as mean ± standard deviation. Statistical evaluation was by one way analysis of variance (anova) between drug treatment groups. This was followed by a post-hoc test (Boneferroni, Tuckey). 95% confidence limits for the difference between groups have been quoted for selected parameters. The null hypothesis was rejected at *P* < 0.05.

## Results

### General

The clinical and baseline anthropometric, demographic and some echocardiographic data in the three different antihypertensive pharmacotherapeutic groups are shown in Table 1. The 3 different treatment groups were well matched in the severity of left ventricular hypertrophy (LVMI), age, blood pressures, gender (but with a slight male proportion in the combination group) and heart rate. None of the patients had symptomatic systolic dysfunction, and the ejection fractions were comparable, with no statistically significant difference between groups. Some data were omitted where echocardiographic findings because they were unrecorded because of unsatisfactory technical quality.

### Diastolic Function

Diastolic dysfunction was assessed by mitral E/A velocity ratio <1, A wave velocity time integral greater than 5 cm, and prolonged IVRT > 100 ms. The association of diastolic function with antihypertensive pharmacotherapeutic class is shown in Table 1.

#### E/A Velocity Ratios

There was a significant class difference in the mitral E/A velocity ratios (*P* = 0.048, anova, *F* = 3.29) 37.% (5/13) of patients receiving enalapril or lisinopril (group A) had E/A ratio > 1. The 95% CI for E/A ratio in this group were 0.8 to 1.33. By contrast, only 8.4% (1/12) of patients receiving calcium channel blockers, amlodipine/ sustained release nifedipine, (group C) had E/A ratio >1. The 95% CI for the E/A in the calcium antagonist group were 0.63 to 0.86. This value was significantly lower than the ACE-inhibitor group. (See Table 1). Combined treatments (A + C) gave E/A values that were intermediate between either groups. See Table 1. 25% (4/16) had E/A ratios > 1. None of the patients in any group had “pseudonormalization”, as all those with E/A ratio > 1, had E wave deceleration time >200 ms.

A wave velocity time integral (AVVTi): The AVVTi was grossly increased in these groups of chronic hypertensives. (See Table 1). There were significant between group differences in this parameter according to antihypertensive pharmacotherapeutic class. ACE inhibitor class alone; 21.9 (4.77) cm, and ACE-inhibitor combined with CCB 20.1 (3.6), had significantly lower AVVTi values than Calcium channel blockers alone 25.3 (6.36). (*P* = 0.037, anova, *F* = 3.613, df = 36).

#### Intraventricular Relaxation Time (IVRT)

These results are shown in Table 1. There was a highly statistically significant difference among the treatment groups (*P* = 0.013 anova, *F* = 4.915, df = 36). In the ACE-inhibitor group (A), 4/13 or 31% had IVRT < 100 ms. The 95%CI for IVRT in this group being 101–129 ms. The Calcium channel blocker group had 2/11 or 18% of its patients with IVRT < 100 ms. Patients on combined ACEI + CCB (A + C) 9/13 or 70% had a normal IVRT < 100 ms. The 95% CI were 82 to 104 ms, which is compatible with the normal range.

### Left Ventricular Hypertrophy and Systolic Contractility Indices

These results are summarized in Table 1. There were no statistically significant differences between groups in LVM, or LVMI or RWT. However, the ACEI group tended to have a lower LVMI of 125(35) g/m^2^compared to the other two groups.

The systolic contractility indices measured as fractional shortening or ejection fraction were all within the normal range in the population, with no between group differences.

## Discussion

Our study was a retrospective analysis of patients who had received treatment for 3–6 months before echocardiographic study, and thus this study is preliminary and only associates the echocardiographic changes with specific pharmacotherapy, but cannot establish a causal link. The three groups of patients appeared well matched in clinical or epidemiological features (Table 1).

The major differences observed between the groups were in the diastolic function. Treatment with ACEI was associated with a higher E/A ratio, such that 37% had values greater than 1 with the E-deceleration time >200 ms. By contrast only 8.4% of CCB recipients had E/A greater than 1, but the combination of ACEI + CCB had E/A ratio of >1 in 25% of the people. With regard to the AVVTi, all the hypertensive patients demonstrated increased values. However, in a similar manner to E/A ratio, AVVTi on ACE-I alone 21.4 (4.77), or ACEI-CCB combination 20.1 (3.6) were significantly lower than CCB alone, 25.3 (6.36) (*P* = 0.037, anova). Prolonged IVRT is another indication of impaired diastolic relaxation, and is abnormal if >100 ms. In these patient groups, 31% of ACEI, and 70% of ACEI + CCB combination had IVRT < 100 ms, compared with only 18% on CCB alone.

Our results indicating salutary effects associated with ACE-inhibitors or calcium channel blockers on diastolic function accords with findings in hypertensives, who were studied before and after repeated echocardiographic measurements [20,25,26]. The additive benefit of CCB with ACEI, especially with regard to the IVRT and AVVTi accords with the notion that ACE-inhibitors improve cardiac relaxation, and that concurrent calcium channel blockade with ACEI may be superior to CCB alone in our population. These results further suggest the combined role of the renin–angiotensin system and calcium ions, in the process of active diastolic relaxation [27].

Previous reports indicate that left ventricular mass and blood pressure affect the IVRT [17,28] but the difference in IVRT in this study is not accounted for by differences in LVMI or blood pressures (Table 1). The apparent beneficial effect of ACEI may be because these drugs have been shown to reduce myocardial fibrosis and/or hypertrophy, despite minimal blood pressure change [20,29]. This is concordant with the notion that diastolic dysfunction may predate or be independent of systolic function in hypertensive heart disease [30].

### Limitations of the Study

This study was retrospective in design, and the numbers of patients were limited.

It is therefore unable to make a definitive causal or mechanistic relationship, between drug class and improvement in diastolic function at a given level of blood pressure or systolic ejection fraction. Such information can only be provided by future prospective and randomized blinded end point study. However, this study provides a preliminary evidence associating ACEI (with or without calcium antagonists) with a better diastolic function.

In conclusion, our preliminary study indicates association between treatment with ACE-I (with or without CCB) with a background of diuretic therapy, and significantly better indices of diastolic function, in a group of Nigerian essential hypertensive patients with left ventricular hypertrophy.
